# Various Techniques of Mechanical Retention in Hand Prostheses and Complex Finger Prostheses: A Case Series

**DOI:** 10.7759/cureus.88408

**Published:** 2025-07-21

**Authors:** Jitendra Jethwani, Lovely M Annamma, Biji Thomas George, Fatma M Hamed AL-Ameen, Chandrasekharan Nair

**Affiliations:** 1 Basic Sciences, Virginia Commonwealth University, School of Dentistry, Richmond, USA; 2 Clinical Sciences, College of Dentistry, Ajman University, Ajman, ARE; 3 General Surgery, Ras Al Khaimah (RAK) Medical and Health Sciences University, Ras Al Khaimah, ARE; 4 Prosthodontics, Sri Sankara Dental College, Trivandrum, IND

**Keywords:** complex finger prosthesis, hand prosthesis, hollow acrylic resin prosthesis, mechanical retention, silicone lining

## Abstract

The hand is associated with both form and function. The loss of a body part, such as a finger or a partial hand, has a profound psychological impact on the patient and significantly reduces their quality of life. A precisely fitting hand prosthesis or complex finger prosthesis can significantly improve function by restoring esthetics, normal length, and function to a great extent, as well as protecting the remnant sensitive stump (if lost due to amputation). Based on their functional capabilities, several types of hand prostheses can be designed, including passive, externally powered, hybrid, and bionic. An implant hand prosthesis is also an option, as it provides a more secure retentive fit. A passive prosthesis is purely cosmetic and does not offer any movement but can be used to stabilize or, at times, carry objects, which could mean a great deal to a person. Most patients prefer the simple glove-type prosthesis with a soft inner lining, primarily driven by affordability, which is held in place by various mechanical retentive means that aid in both function and cosmetic appearance. This article presents a case series of partial/complete hand prostheses. The uniqueness of this case series lies in the fact that various mechanical retentive methods are employed in each case type to achieve both function and appearance. The first case involves a complete hand prosthesis fabricated as a 'hollow' glove-type hand prosthesis, which aims to reduce the weight and, consequently, the cost of the prosthesis. The other case reports involve complex finger prosthesis cases with mechanical aids, such as finger rings and silicone linings, which provide improved comfort and acceptance. It also enables the colour matching of the dorsal and ventral surfaces separately, so that the prosthetic form, colouration, and texture are as indiscernible as possible from the surrounding tissues.

## Introduction

The development of hands takes place in the fourth to sixth weeks of pregnancy in utero [1]. Genetic or environmental factors can influence the normal development and growth of the arms, hands, and fingers within the womb. Loss of arms or the hand portion alone can be due to congenital malformations such as polydactyly or syndactyly [2]. Other deformities include cleft hands, which occur in 2-4% of infants born with congenital abnormalities of the hands [3]. Symbrachydactyly is a congenital deformity of the hand and fingers in which the infant is born with completely missing or small, underdeveloped fingers. The moderate expression of this defect occurs when the baby is born with small projections of soft tissue covered by the skin for the fingers and a shorter thumb than normal. It can also manifest as webbed fingers (syndactyly) [4]. Apart from congenital malformations, the next common cause of the loss of a hand is trauma. The number of amputations due to diabetes or malignant diseases is lower than that caused by trauma. Individuals who have lost a hand are psychologically affected because their day-to-day activities are disrupted, and their everyday life is affected. Hence, an early replacement with a prosthesis is necessary [5]. Prosthetic replacement could psychologically benefit the person who has lost a hand or digits because of trauma [6].

The options for lost hand replacement range from a simple prosthesis to the surgical reattachment of a severed finger or hand to a complicated reconstructive human hand transplantation. The simplest esthetic prosthesis is a glove-like contrivance fabricated in silicone or acrylic material [7]. While the silicone material mimics the natural appearance, texture, and colour of a biological human hand, a hollow acrylic prosthesis is a better choice to reduce its weight and cost. Both of these materials are used as maxillofacial prosthetics [8]. The added advantage of hollow acrylic prosthesis is that it holds its shape well and can provide excellent cosmetic results [9].

The authors present a case series of complete and partial hand prostheses in this article. One is a full-hand prosthesis with a Velcro attachment for better retention; the other two case reports feature complex finger prostheses, restored with an intrinsically coloured hollow acrylic prosthesis fitted with a finger ring, and one with a silicone liner. The silicone liner provides a softer feel, acts as a shock absorber, and is biocompatible. Both prostheses were fabricated following the same steps and method. The aim was to deliver a lightweight, colour-matched hand prosthesis to the patient's satisfaction. Intrinsic colouration was preferred based on reported literature, which shows that the prosthesis changes colour over time with extrinsic colouration techniques [8].

## Case presentation

Case 1

A 27-year-old healthy male with an amputated right hand was reported for a full-hand prosthesis. The amputation was at the level of the wrist and was caused by a motorbike accident. Clinical examination elicited no tenderness or evidence of bony spicules on palpation. The stump looked well-healed post-surgically. A radiographic examination revealed a healthy remnant bone structure. This patient also refused any further surgery to place implants and chose the simple hollow acrylic prosthesis, as it was the only option he could afford. A cuff-like attachment was selected to retain the prosthesis on the stump. Skin colour and shade matching was performed meticulously using the normal left hand as the guide. The inner layer was lined with silicone for a tighter fit.

Clinical steps for hollow hand prosthesis

**Figure 1:**
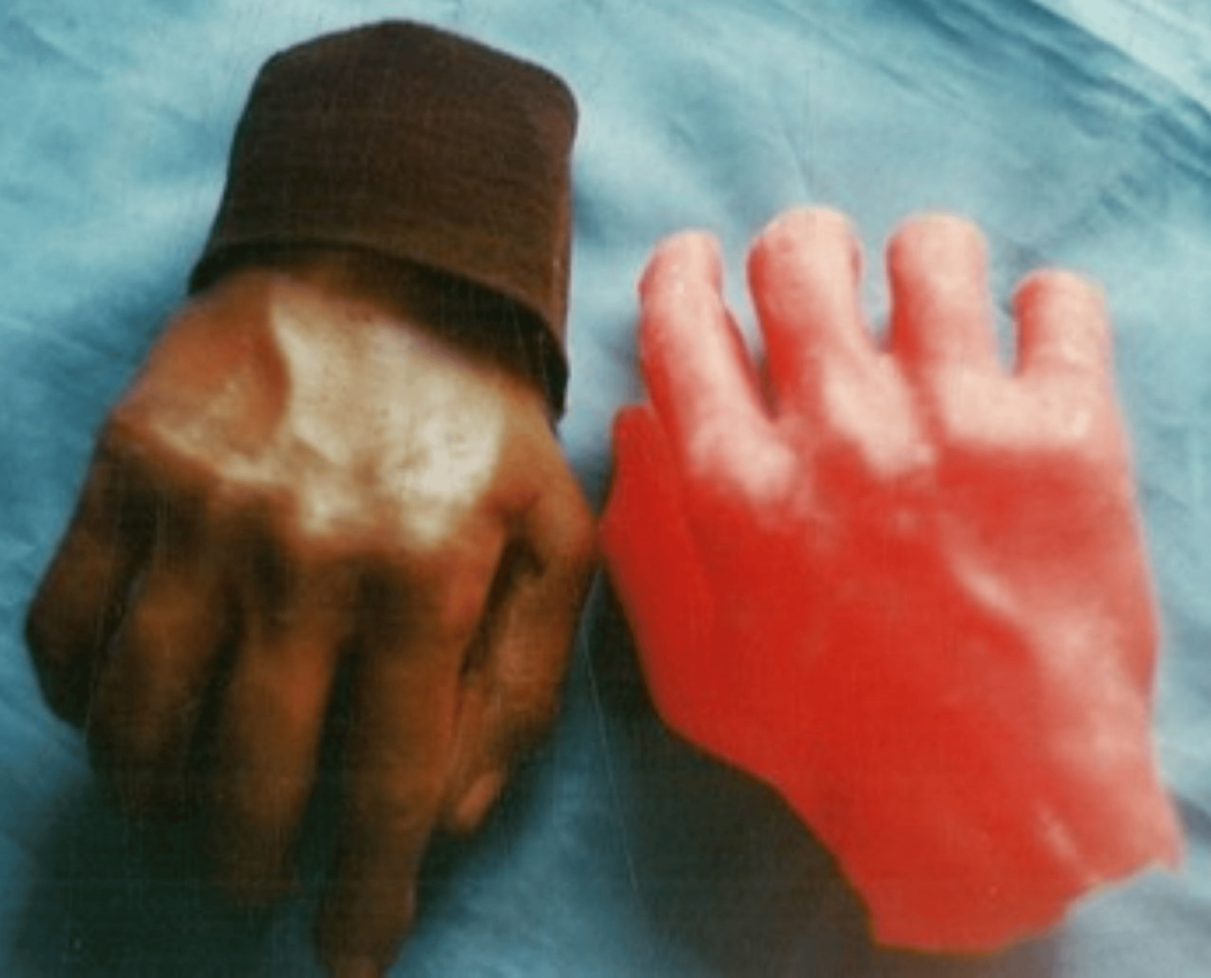
Wax pattern of a full-hand prosthesis

An impression of both hands was made using alginate impression material (Jeltrate, Dentsply Sirona, Charlotte, NC, USA), after lubricating the hands with petroleum jelly to facilitate easy removal. The hand was held loosely over a square tray, and the impression was painted on all surfaces, carefully avoiding any air bubbles being trapped. Cotton shreds were spread on the setting alginate, and a layer of gypsum was patched onto the alginate impression to retain it in its exact form and shape. The impression of the deformed hand was poured into dental stone (Kalstone), and a replica model was obtained. The other hand impression was used to make a wax pattern (modelling wax) in a hollow form by coating the inner surface of the impression with wax (Figure 1).

**Figure 2:**
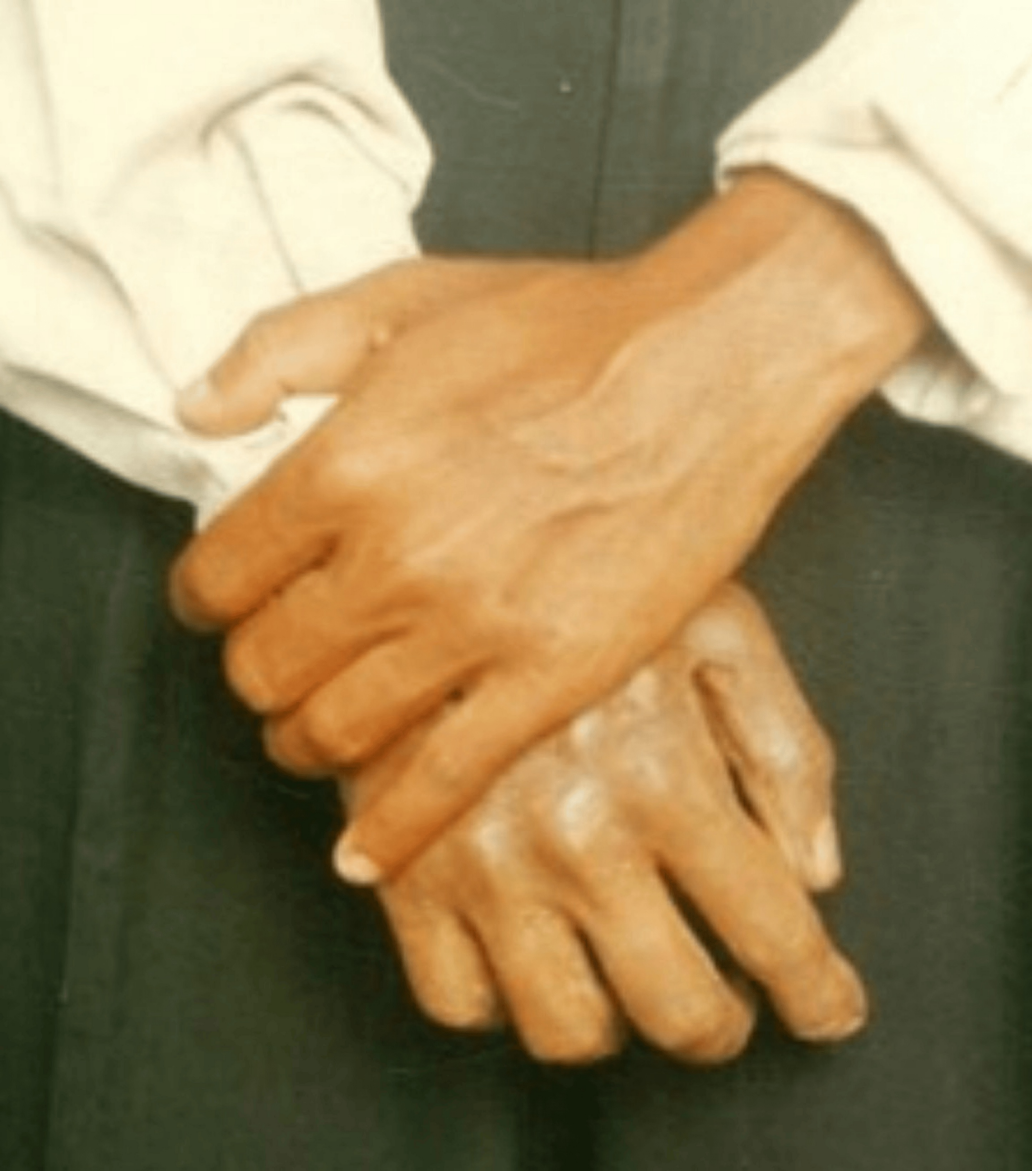
Final full-hand acrylic prosthesis colour-matched to the patient's skin tone

The retrieved wax pattern was inspected for any deformation and air bubbles. The wax pattern was tried, carved, and shaped to the patient's satisfaction before finalization. After finalizing the trial prosthesis for the fit check and cosmetic appeal, the wax pattern was invested in a split mould technique. The ventral and dorsal halves were separately duplicated in dental stone (white Kalstone). A patch test was done to eliminate allergies to acrylic resin. The shade matching was done using a chair-side trial-and-error technique with pigments and dyes in pink acrylic. The shade match was approved by the patient and was set aside to be used as the shade guide. Colours were mixed in the polymethyl methacrylate (PMMA) monomer, which was added to the pink PMMA powder for the dorsal and ventral surfaces. The already colour-matched pigment combination was added to the PMMA monomer and then mixed with clear PMMA powder (cold-cure PMMA DPI). After the ventral and dorsal halves were colour-matched, the acrylic prosthesis was retrieved and joined by more colour-matched acrylic. A pre-prosthetic final prosthesis try-in was done. The patient complained about the retention; an additional retentive Velcro was attached (Figure 1). The final prosthesis was checked for fit, comfort, and patient satisfaction (Figure 2). The patient was very satisfied and appreciated the time and effort taken to obtain a prosthetic hand for him. The patient communicated several times over a couple of years, sending more cases, and even stated that he sometimes used it to ride his motorbike.

Case 2

A 35-year-old male patient, otherwise normal-looking and in good health, reported a factory accident and losing two digits on his left hand, the ring finger and the small finger. He was working as a mechanic and, due to the loss of digits, faced psychological distress and desperately wanted a cosmetic restoration to cover the defect. After considering various treatment options, including implant-retained, functional, and silicone esthetic prostheses, he opted for the hollow acrylic prosthesis due to financial reasons. The merits and demerits of an acrylic prosthesis, including discolouration and the occurrence of a tapping sound when contacting other solid objects, were clearly explained to the patient.

**Figure 3:**
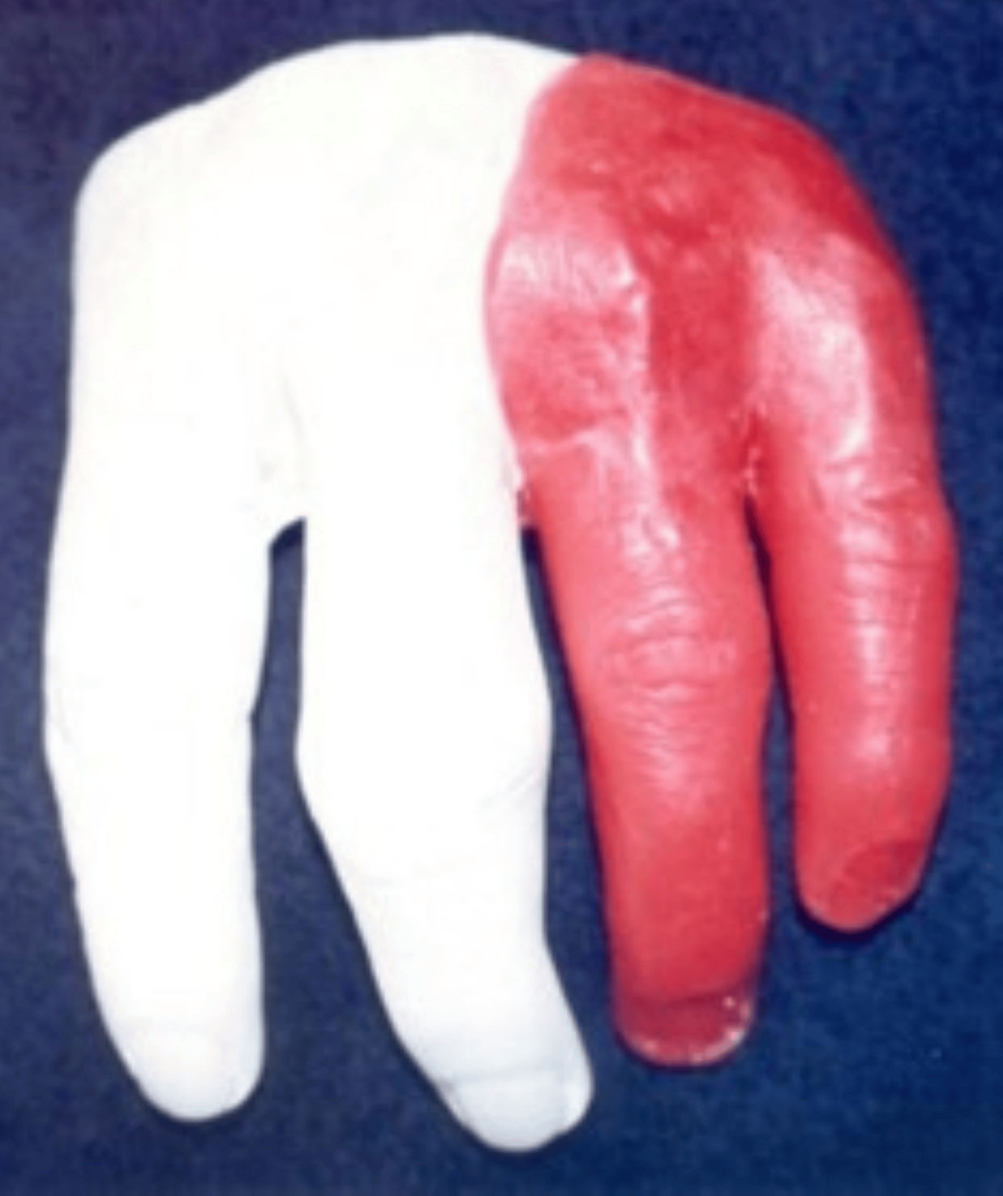
Wax pattern of a two-digit finger prosthesis

**Figure 4:**
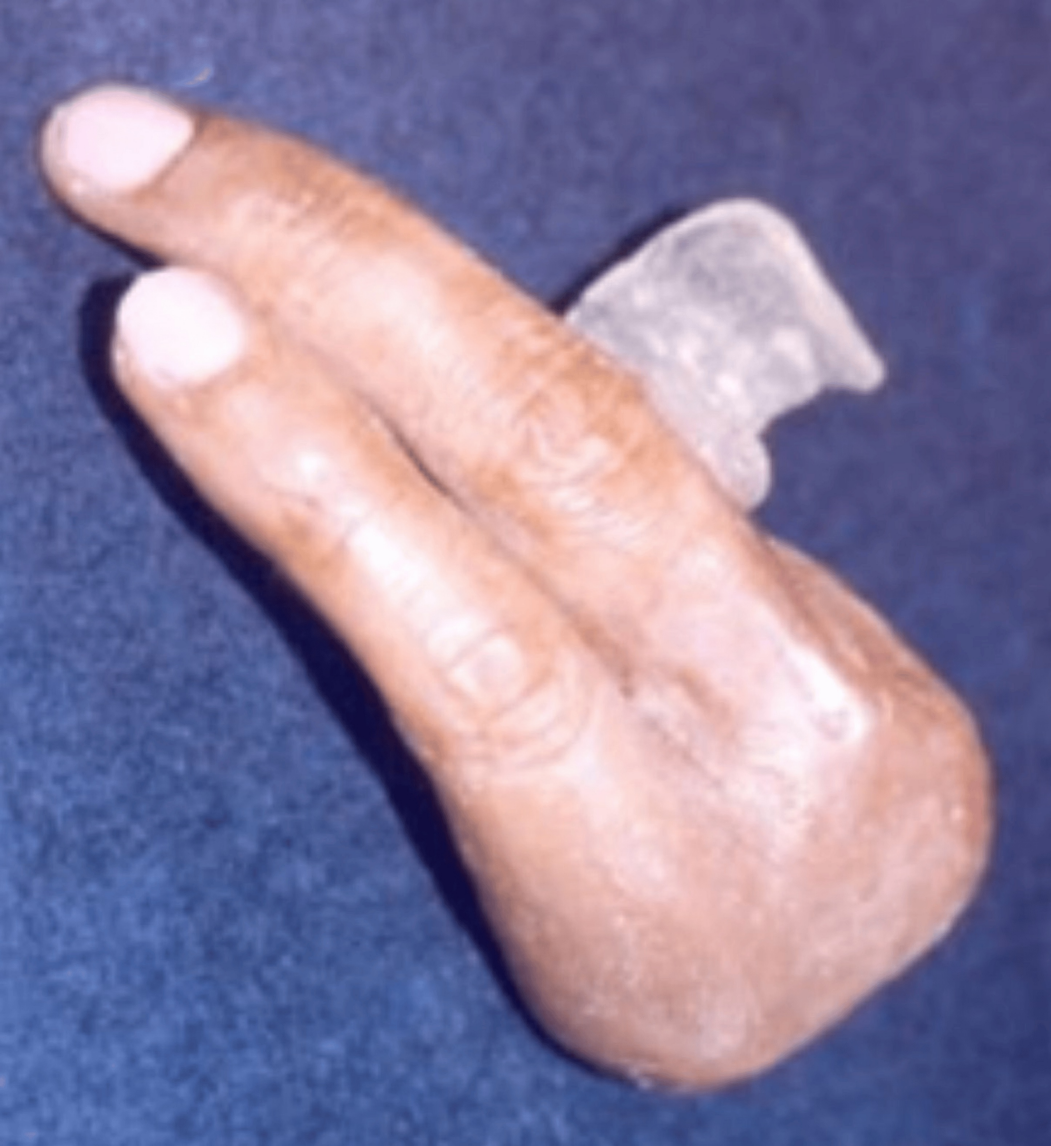
Final prosthesis of two digits with acrylic lock support

**Figure 5:**
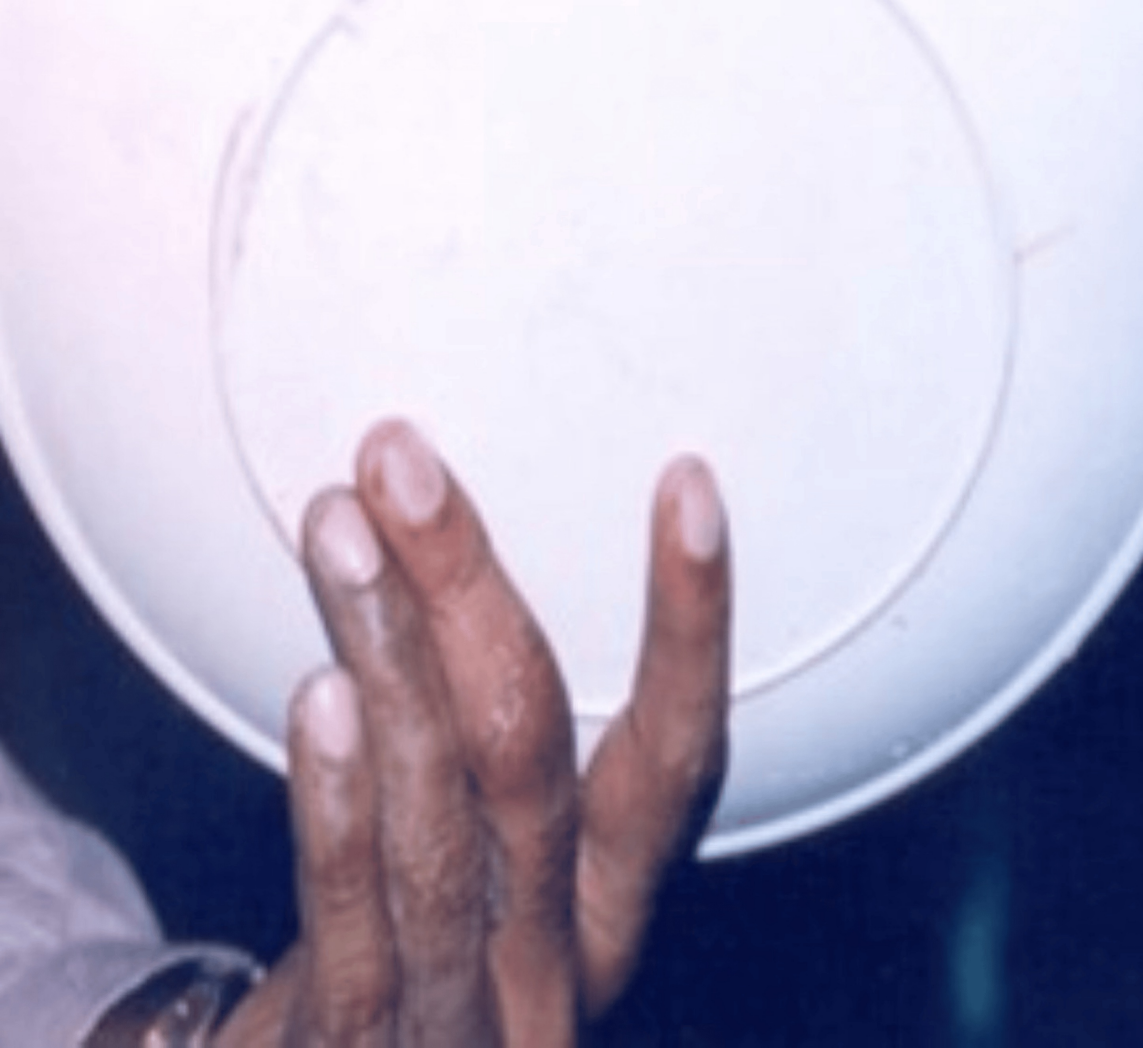
Attached finger prosthesis during function

Clinical examination of the deformed hand showed that the excision had no stump support, and retention was from the adjacent finger. The patient refused external appearance support; hence, the prosthesis was extended to the dorsum of the hand, and support was placed on the ventral of the adjacent middle finger. An inner soft lining with silicone was provided for added comfort and a secure fit. No pain or tenderness was elicited on palpation of the fingers. The radiographic examination did not reveal any defects. Careful shade selection was done using pigments (Factor II Inc., Lakeside, AZ, USA), matching the dorsal and ventral sides separately (Figures 3-5).

Case 3

**Figure 6:**
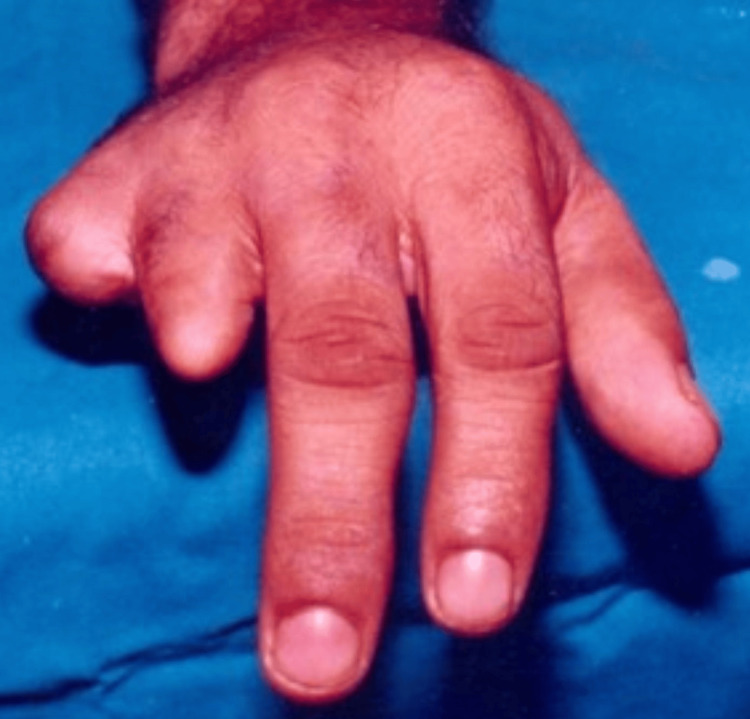
Postoperative finger loss

A 32-year-old male patient, otherwise normal-looking and in good health, reported amputation of two digits in a road traffic accident involving his right ring finger and small finger (Figure 6).

**Figure 7:**
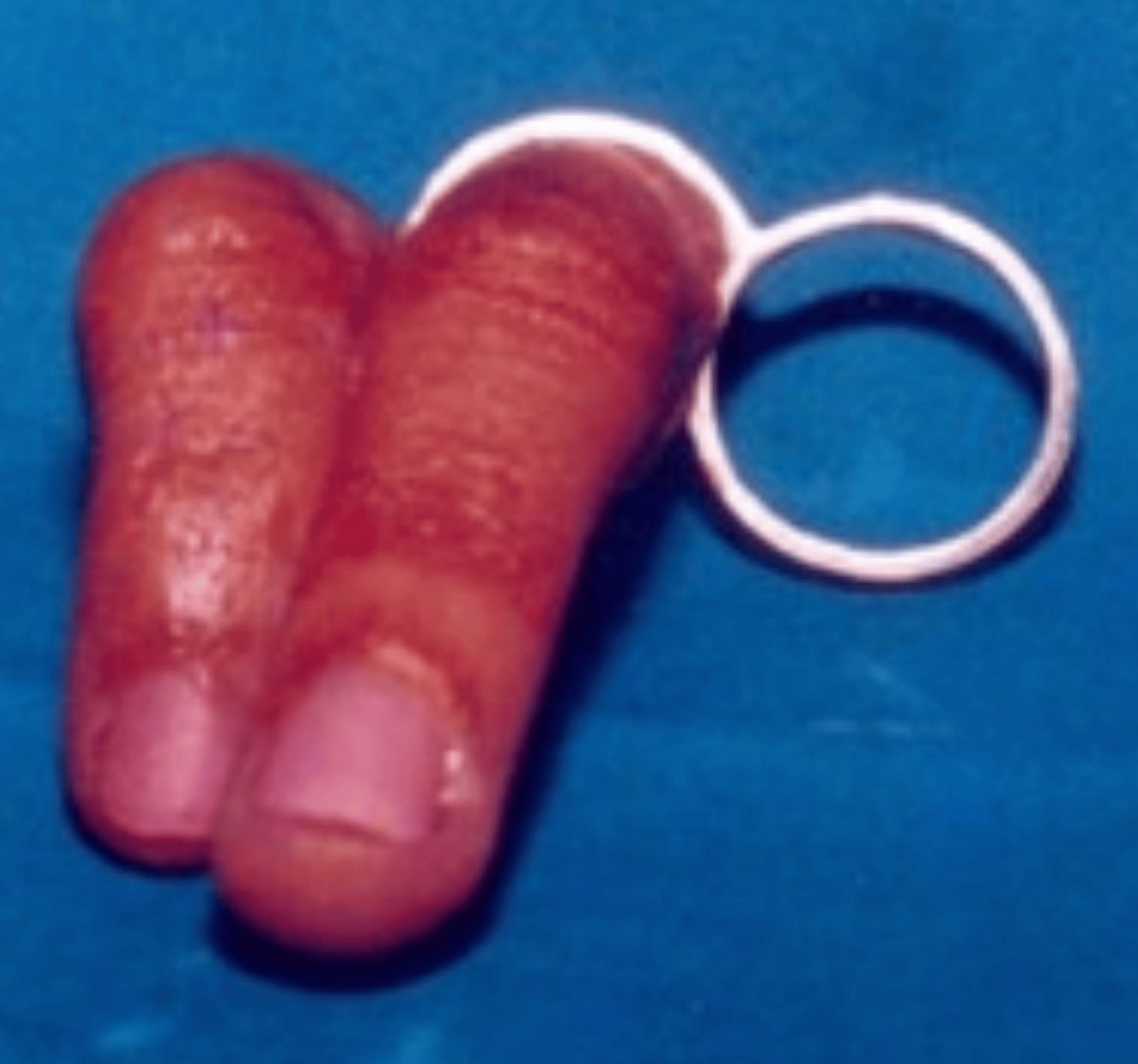
Final finger prosthesis with ring support

**Figure 8:**
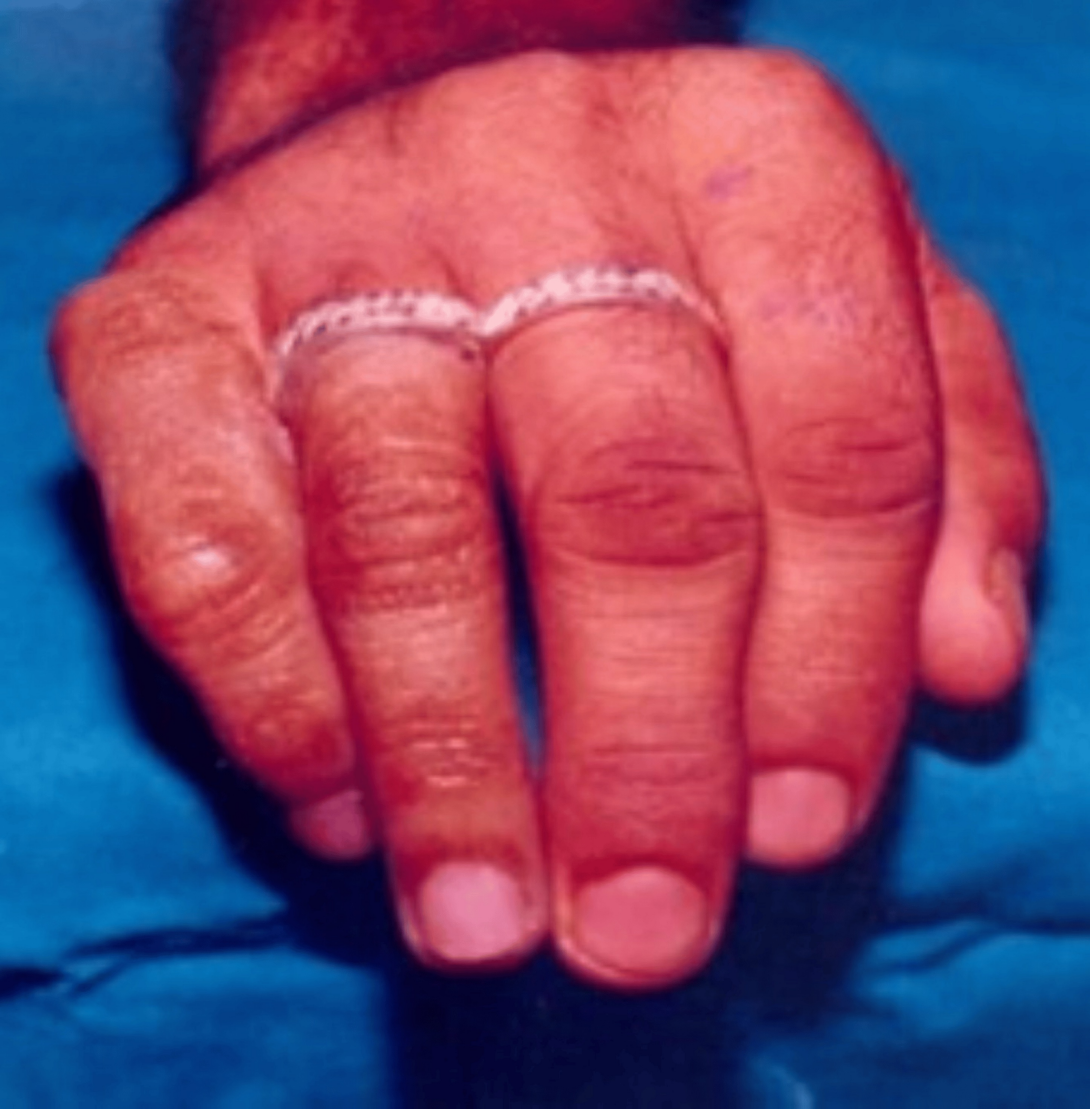
Completed finger prostheses

The stumps were well healed, and he wanted an economical cosmetic restoration. Clinical examination of the deformed hand showed that the remaining finger stump was well healed. The patient opted for a ring-supported acrylic hollow skin-coloured prosthesis. The radiographic examination did not reveal any defects. Careful shade selection was performed using pigments (Factor II Inc., Lakeside, AZ, USA), matching the dorsal and ventral sides separately (Figures 7, 8).

Clinical steps for finger prostheses of Cases 2 and 3

An impression of the finger stump area was made using alginate impression material (Jeltrate, Dentsply Sirona, Charlotte, NC, USA) after lubricating the area with petroleum jelly to facilitate easy removal of the impression. The hand was held loosely over a square tray, and the impression was painted on all surfaces. Cotton shreds were spread on the setting alginate, and a layer of gypsum was patched onto the alginate impression to retain it in its exact form and shape. The impression of the deformed hand with two digits missing was poured into dental stone (Kalstone), and a replica model was obtained. The other hand finger impression was used to make a wax pattern (modelling wax) in a hollow form by coating the inner surface of the impression with wax. The retrieved wax pattern was inspected for any deformation and air bubbles.

Before finalization, the wax pattern was tried, carved, and shaped to the patient's satisfaction. After finalizing the trial prosthesis fit check and cosmetic appeal, the wax pattern was invested in a split mould technique. The ventral and dorsal halves were separately duplicated in dental stone (white Kalstone). A patch test was conducted to identify and eliminate potential allergies to acrylic resin. The shade matching was done using a chair-side trial-and-error technique with pigments and dyes in pink acrylic. The shade match was approved by the patient and was set aside to be used as the shade guide. Colours were mixed in the PMMA monomer, which was added to the pink PMMA powder for the dorsal and ventral surfaces. The already colour-matched pigment combination was added to the PMMA monomer and then mixed with clear PMMA powder (cold-cure PMMA DPI). After the ventral and dorsal halves were colour-matched, the acrylic prosthesis was retrieved and joined by more colour-matched acrylic. A pre-prosthetic final prosthesis try-in was done. In Case 2, additional retentive plate support was used on the ventral side of the ring finger (Figure 4). A silicone liner with adhesive on the inner side was used for a tighter fit (Factor II Inc.). In Case 3, additional rings were used for support and retention (Figure 6). The final prosthesis was checked for fit, comfort, and patient satisfaction.

## Discussion

The fundamental reason a patient seeks a prosthetic replacement for any lost or missing body part is the cosmetic factor, causing emotional distress, lack of confidence in social meetings, and limited functional range of the hand. The most common materials used in the fabrication of hand prostheses are silicones. Medical-grade silicones give a life-like appearance and feel to the prosthesis. In this case series, the patients chose acrylic resin instead of silicone due to its affordability. It is also the easiest to fabricate and the most economical. Additionally, an acrylic hand prosthesis with an inner silicone lining to protect the stump is reasonably comfortable to wear. Acrylic resins are more colour-stable, able to withstand rough use, and easily repairable [10]. Based on the patient's request for an economical and long-lasting prosthesis, acrylic material was selected. In one case, a silicone liner was used inside in contact with the stump. The silicone liner was attached to the acrylic by an adhesive primer (soft reline primer, Tokuyama Dent Corp., Japan) using an open-vent curing method [11]. Both patients were followed up after six months by phone. Both were satisfied and continued to use the prosthesis.

Technological advancements offer many improved and functional options for hand prostheses. A body-powered partial hand prosthesis utilizes the movements of the remaining portions of the upper body, hand, or arm to provide some function. More sophisticated options include the electrically powered partial hand prostheses, activity-specific partial hand prostheses, or hybrid prostheses that combine two or more of the above-mentioned prosthetic alternatives (Arm Dynamics) [12]. The myoelectrically controlled prosthesis has functions enabling the hand to move. Electrodes positioned within the prosthesis sense muscle movement and transmit it as electronic signals to electrodes within the prosthesis, causing the movement of the hand. These are expensive and need proper maintenance [13]. The other types are self-powered, utilizing principles of mechanics, and hybrid prostheses (a combination of self-powered and myoelectrical) [14].

## Conclusions

Considering the number of patients losing their hands due to cancer, trauma, or congenital deformity, the diverse options available are advanced. The choice of prosthesis depends on the patient's needs. Patients request simple prosthetic replacements for cosmetic appeal. However, myoelectric prosthetic replacements can enable function to a considerable extent. The major drawback is their cost. More advanced technologies, such as self-healing sensors on hand prostheses, are being discussed and are in their initial phases. In these three cases, an acrylic and, in one case, acrylic with a silicone liner achieved a good cosmetic factor and patient satisfaction.
